# Tuning Curves, Neuronal Variability, and Sensory Coding

**DOI:** 10.1371/journal.pbio.0040092

**Published:** 2006-03-21

**Authors:** Daniel A Butts, Mark S Goldman

**Affiliations:** **1**Division of Engineering and Applied Sciences, Harvard University, Cambridge, Massachusetts, United States of America; **2**Wellesley College, Department of Physics and Program in Neuroscience, Wellesley, Massachusetts, United States of America; Montana State UniversityUnited States of America

## Abstract

Tuning curves are widely used to characterize the responses of sensory neurons to external stimuli, but there is an ongoing debate as to their role in sensory processing. Commonly, it is assumed that a neuron's role is to encode the stimulus at the tuning curve peak, because high firing rates are the neuron's most distinct responses. In contrast, many theoretical and empirical studies have noted that nearby stimuli are most easily discriminated in high-slope regions of the tuning curve. Here, we demonstrate that both intuitions are correct, but that their relative importance depends on the experimental context and the level of variability in the neuronal response. Using three different information-based measures of encoding applied to experimentally measured sensory neurons, we show how the best-encoded stimulus can transition from high-slope to high-firing-rate regions of the tuning curve with increasing noise level. We further show that our results are consistent with recent experimental findings that correlate neuronal sensitivities with perception and behavior. This study illustrates the importance of the noise level in determining the encoding properties of sensory neurons and provides a unified framework for interpreting how the tuning curve and neuronal variability relate to the overall role of the neuron in sensory encoding.

## Abstract 2

This study provides a unified framework for interpreting how a neuron's stimulus tuning curve and response variability relates to sensory encoding.

## Introduction

Since the earliest studies of sensory systems [ 1], the contribution of individual neurons to sensory function has been assessed by measuring their responses to a relevant set of stimuli. The standard tool in performing this characterization is the neuronal tuning curve: a plot of the average firing rate of the neuron as a function of relevant stimulus parameters. Tuning curves have provided the first-order description of virtually every sensory system, from orientation columns in the vertebrate visual cortex, to place cells in the hippocampus and wind-detecting neurons in the cricket cercal system [ 2]. Despite their ubiquitous application and straightforward formulation, the interpretation of tuning curves remains an issue of debate.

The most common interpretation of tuning curves is that the stimuli at their peak, which evoke the highest firing rates **,** are most important to a neuron. For example, orientation columns in the visual cortex are typically labeled by the orientation that evokes the most activity at each location, effectively identifying each neuron with a single stimulus at its tuning curve peak. Such a reduction has strong intuitive appeal because high firing rates are most distinguishable from background firing and other noise in the system.

In contrast, many studies have noted that nearby stimuli are most easily discriminated in high-slope regions of the tuning curve, because in these regions, small changes in the stimulus result in the largest changes in firing rate [ 2, 3]. From this perspective, the peak of the tuning curve is a particularly insensitive region of the neuron's response because the slope at the peak is zero.

Here we show that both interpretations can be correct, depending on the amount of neuronal variability and the experimental context. Low levels of neuronal variability favor sensitive or “fine” discrimination of nearby stimuli in regions of high slope. However, when response variability increases, fine discrimination becomes disrupted and responses can only be reliably distinguished on a “coarser” scale. In this case, high-firing-rate responses stand out, making stimuli at the peak of the tuning curve most distinguishable. We demonstrate this intuition by applying three different information-based measures of stimulus encoding to experimental characterizations of sensory neurons, and demonstrate a transition from “high-slope” to “peak-firing-rate” encoding as neuronal variability increases, both for single neurons and in the context of a small population.

These measures also can be applied to experiments that probe how individual neurons contribute to perception in different experimental contexts. We consider recent experiments correlating neuronal activity the medial temporal cortex (area MT) to performance during two-alternative discrimination tasks [ 4, 5], and show how the different tasks used in these studies either isolate the effects of “high-slope” encoding [ 5] or “peak-firing-rate” encoding [ 4]. Thus, we provide a consistent framework for interpreting previous experimental and theoretical results, and demonstrate the importance of considering neuronal variability in determining the role of neurons in sensory processing.

## Results

### Defining the Information Associated with Particular Stimuli

**Figure 1:**
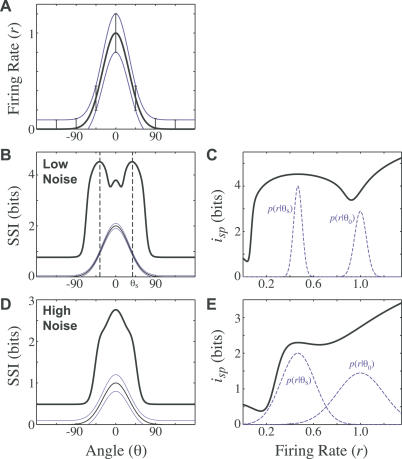
Illustration of Slope-to-Peak Transition in the SSI with Increasing Level of Neuronal Variability (A) Typical tuning curve of a neuron, with mean firing rate (thick line) and standard deviation (thin lines) shown as a function of the stimulus parameter θ. These are reproduced as thin lines for reference in (B) and (D). In this example, the standard deviation of the firing rate for a given value of θ increases with increasing firing rate from a baseline value, although the particular form of noise chosen does not qualitatively affect our results. (B and D) The SSI(θ) is maximum in regions of high slope in the low-noise case (B), and maximum at the tuning curve peak in the high-noise case (D). (C and E) The specific information (solid line) in the low- and high-noise cases shown as a function of normalized firing rate. *p*( *r*|θ) is shown for reference at θ *_S_* (left) and θ _0_ = 0 (right).

To illustrate the effect of noise on the encoding of stimuli, consider the canonical example of a sensory neuron characterized by a bell-shaped tuning curve function *f*(θ), representing the average firing rate of the neuron as a function of a stimulus parameter θ ( Figure 1A, thick line). Across multiple presentations of the same stimulus, the neuron will have a distribution of responses sampled from *p*( *r*|θ): the probability of *r* given stimulus θ. The width of this distribution represents the neuronal variability ( Figure 1A, error bars and lines). Neuronal variability can be due to factors such as intrinsic noise, integration time of the neural response, and aspects of the stimulus not represented by the parameter θ (see Materials and Methods and Protocol S1).

How can variability affect how well a stimulus is encoded by a neuron? Intuitively, a stimulus is well encoded if it evokes unambiguous responses [ 6]. An unambiguous response is one that could only be evoked by a small number of stimuli so that the stimulus is readily identified when this response appears. For example, a modest firing rate might unambiguously represent a particular stimulus when there is no background activity, but will become more ambiguous as background activity increases.

This intuition can be formalized by the specific information of a response [ 7]:



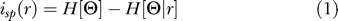



defined as the difference between the entropy of the stimulus ensemble *H*[Θ] = − ∑ _θ_ *p*(θ) log _2_ *p*(θ) and that of the stimulus distribution conditional on a particular measurement *H*[Θ| *r*] = − ∑ _θ_ *p*(θ| *r*) log _2_ *p*(θ| *r*). Since the entropy of a stimulus distribution is a measure of how uncertain the stimulus is, the specific information *i*_sp_( *r*) gives the reduction in uncertainty about the stimulus gained by a particular response *r,* and thus is high for unambiguous responses and low for ambiguous responses.

Well encoded stimuli can thus be identified by their association with unambiguous responses as defined by *i*_sp_( *r*). We therefore use the stimulus-specific information (SSI) [ 6] as our measure of stimulus encoding:



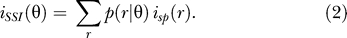



The SSI is the average specific information of the responses that occur when a particular stimulus θ is present.

The results described below using the SSI are calculated numerically for a given tuning curve and model of variability (see Materials and Methods). Although we focus on the SSI metric because of its straightforward interpretation in terms of the relationship between stimulus and response in encoding, the results described below are also obtained with other information-based metrics, as demonstrated in Protocol S2.

### Transition in the Best-Encoded Stimulus from High-Slope to High-Firing Rates for a Single Neuron

We first compute the SSI for a neuron with a typical bell-shaped tuning curve, as shown in the example of Figure 1A. In the case of low noise ( Figure 1B), where the firing rate variability is a small fraction of the mean firing rate, we find that the maximum SSI does not occur at the peak of the tuning curve θ _0_ = 0. Rather, the best-encoded stimuli occur at ±37° ( Figure 1B, dashed lines), close to the maximum slope of the tuning curve.

This result suggests that the regions of high slope are the most significant to the neuron. However, two points are of note: (1) the highest SSI is close to, but not directly at, the point of maximum slope, and (2) there is a smaller peak in the SSI at the tuning curve peak θ _0_ where the slope of the tuning curve is zero.

To gain a better understanding of this result, the specific information associated with the underlying neural responses ( *i*_sp_( *r*), Equation 1) is shown in Figure 1C. The specific information is largest for responses triggered by relatively few stimuli. Since the number of stimuli associated with a given response scales with the reciprocal of the tuning curve slope, responses in high-slope regions of the tuning curve will typically have higher specific information, resulting in the broad peak in specific information for intermediate firing rates ( Figure 1C).

However, consideration of the slope alone does not determine the specific information. In fact, the specific information is largest for firing rates greater than one (where one corresponds to the maximal firing rate in the absence of noise [ Figure 1A]). Such rates have the largest specific information because very few stimuli are associated with them, since the only way these can occur is when noise increases an already high firing rate. At the same time, the least specific information is associated with low firing rates. This reflects that low firing rates may be “background firing,” since they can be generated by any stimulus in the flat “non-tuned” portion of the tuning curve. Thus, low firing rates correspond to a wide range of possible stimuli and, correspondingly, to low specific information. This occurs despite the fact that the low-firing-rate regions of the tuning curve also have the smallest firing-rate variability.

The SSI represents an average of the specific information over the responses associated with a given stimulus *p*( *r*|θ) ( Equation 2): two examples of these distributions are shown in Figure 1C (dashed lines) for the angle of maximum SSI (left) and the peak of the tuning curve (right). In this way, both the large values of the SSI in the high-slope regions of the tuning curve and the local maximum of the SSI at θ _0_ = 0 can be understood ( Figure 1B).

Figure 1D shows the SSI for a neuron with the same tuning curve as in the example of Figure 1B but with 4× the noise level. The stimulus with the highest SSI in this case occurs at the peak of the tuning curve θ _0_ = 0, which can again be understood by considering the underlying specific information of responses ( Figure 1E).

The specific information has changed in two important ways as a result of noise. First, background firing (from non-tuned stimuli) has a farther-reaching effect on low firing rates. More crucially, high firing rates now have the largest specific information due to their relative lack of sensitivity to noise. Whereas an intermediate firing rate can be evoked by stimuli with lower average firing rates (that noise can raise) or stimuli with larger average firing rates (that noise can lower), very high firing rates (at or above the peak average firing rate) can only result from lower firing rates that noise has raised. Furthermore, as noise increases, more firing rates above one are possible, and these rates all preferentially encode the stimuli around the peak of the tuning curve.

To summarize, features of the SSI curves of Figure 1B and 1D can be understood from the underlying specific information of responses. Responses are informative for two separate reasons, which we will refer to as “fine discrimination” and “coarse discrimination.” Fine discrimination involves distinguishing between neighboring stimuli, which is most easily done in high-slope regions of the tuning curve where responses to neighboring stimuli are most distinct. At the same time, high firing rates stand out above all other responses, and thus can be used for coarse discrimination between stimuli around the peak of the tuning curve and all other stimuli. Both types of discrimination are important for identifying stimuli, but the relative importance of these two types of discrimination is influenced by the amount of neuronal variability. As a result, the amount of neuronal variability changes the relative importance of these two effects, with fine discrimination being more important for low noise, and coarse discrimination more important for high noise.

We show in the Supporting Information that the transition from “high-slope/fine-discrimination” encoding at low noise to “peak-firing-rate/coarse-discrimination” encoding at high noise occurs for two other information-based metrics of stimulus encoding ( Protocol S2), and is robust to the exact form of noise model used ( Protocol S1).

### The SSI in Experimentally Characterized Neurons

The preceding example illustrates behavior of the SSI that is representative of many neuronal tuning curves. We next calculate the SSI for two examples of neurons with tuning curves and noise parameters taken from experimental data (see Materials and Methods): orientation-tuned neurons in the primary visual cortex [ 8] and wind-detecting neurons in the cricket cercal system [ 9].

**Figure 2:**
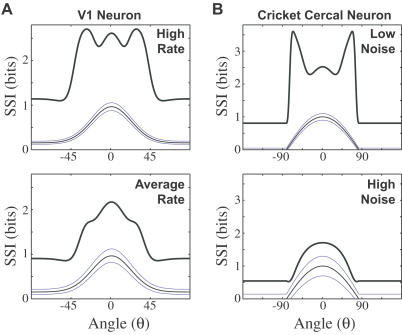
The SSI for Experimentally Measured Neurons (A) The SSI for two orientation-tuned V1 neurons: one with the average firing rate of the studied population (bottom) and one with 3× the average firing rate (top). Since the neuronal variability is given by Poisson statistics [ 8], higher firing rates correspond to lower noise (see text). (B) SSI for a cricket cercal neuron [ 9] for low (1×, top) or high (3×, bottom) noise levels.

Figure 2A shows two examples of V1 orientation tuning curves reported by Kang et al. [ 8] (thin lines) and the resulting SSI. As in the previous tuning curve example, a transition in the maximum SSI is shown with increasing noise level. Neuronal variability in these neurons is described by Poisson statistics [ 8], for which the ratio of the variance in the number of spikes resulting from a given stimulus to the mean number of spikes (known as the “Fano factor”) is one. This implies that the neuronal variability, as measured by the ratio of the standard deviation to the mean number of spikes produced, decreases as 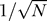, where *N* is the mean number of spikes resulting from each stimulus. Therefore, neurons that produce large numbers of spikes (due to either high firing rates or long counting windows) will have relatively low noise levels. Figure 2A (top) shows such an example for a neuron whose mean firing rate is 3× the average firing rate of the population measured by Kang et al. [ 8]. The SSI peak for this low-noise case is located close to the high-slope portions of the tuning curve ( Figure 2A, top), although the peak at θ = 0 is nearly as large. In contrast, the SSI of a V1 neuron with the mean firing rate is largest at the peak ( Figure 2A, bottom), reflecting that the V1 neurons with average properties of this data set are in the high-noise regime.

We next consider the SSI for a single neuron with parameters corresponding to those measured in the cricket cercal system [ 9]. The cricket has a set of four wind-sensitive neurons whose tuning curves have approximately identical, truncated cosine shape but with peaks offset by 90°. Cosine-shaped tuning curves have been widely used to describe the sensory responses in a wide variety of systems (for a summary, see Salinas and Abbott [ 10]). Unlike the previous example of V1 neurons, the level of variability of these neurons was found to vary linearly with firing rate [ 9] (see Materials and Methods).

Figure 2B (top) shows the tuning curve (thin lines) and the corresponding SSI (thick line) for one such neuron. Consistent with theoretical studies of this neuron [ 10, 11], we find that the maximum SSI does not occur at the peak of the tuning curve θ _0_ = 0. Rather, the stimuli with the largest SSI are located at ±67°, close to the maximum slope of the tuning curve (which in this case is located at the outside edges of the tuning curve).

For higher noise levels, such as might occur for example in less idealized wind conditions, there is again a transition in the peak of the SSI from the maximum-slope to the maximum-firing-rate portions of the tuning curve. For example, when the noise level is increased by 3×, the SSI displays a clear peak at the maximum firing rate ( Figure 2B, bottom).

### SSI for a Four-Neuron Population

Sensory systems commonly mitigate the effects of noise by encoding the same stimulus features with several neurons. Here we calculate the information conveyed about a given stimulus by a small population of neurons and then estimate the contribution of individual neurons to this information to see whether there is again a slope-to-peak transition in the best-encoded stimuli.

**Figure 3:**
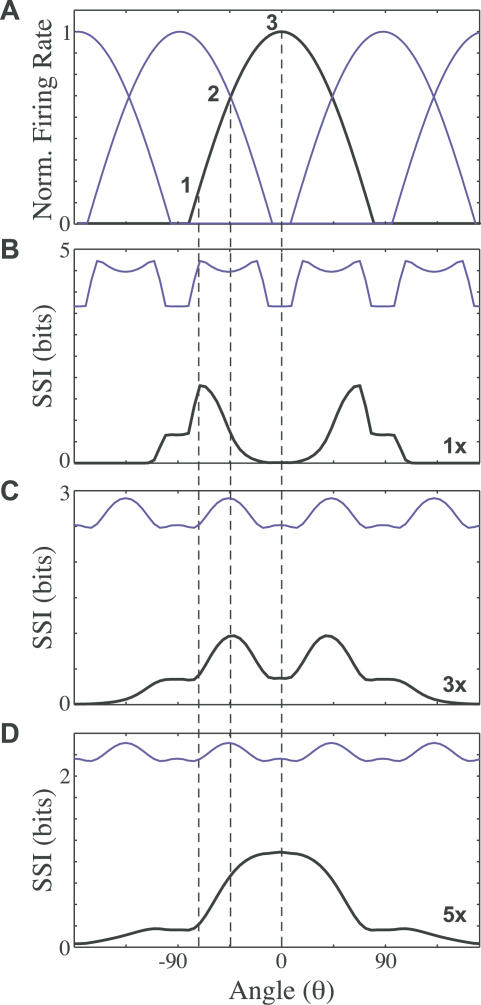
Population SSI and Marginal SSI in the Context of a Four-Neuron Population (A) Tuning curves of the cricket cercal system interneurons studied. (B–D) Population SSI (thin line) and marginal SSI of the center neuron (thick line) for low (1×), medium (3×), and high (5×) noise cases, demonstrating a transition in the maximum marginal SSI from slope (1) to intersection (2) to peak (3).

In the context of a population, the information that each individual neuron contributes might not be the same as the information that this neuron would convey in isolation, because information may be encoded redundantly or synergistically between neurons [ 12]. In this light, the results presented above ( Figures 1 and 2) can be considered as assigning a primary role to the evaluated neuron, so that the role of the remaining neurons in the population is to provide missing information not provided by this first neuron. Below we consider the opposite limit and calculate the “marginal contribution” of a single neuron to the information already provided by all other neurons in the population, i.e., we calculate the difference in population information resulting from removing the neuron from the population. The calculation of the population SSI proceeds identically to that for a single neuron except that the single-neuron firing rate *r* is replaced by a vector of firing rates *r* = { *r*_1_, *r*_2_,…, *r _N_*} representing the responses of each of the *N* neurons in the population. We first calculate this population SSI for the four-neuron cricket cercal population parameterized experimentally by Miller et al. [ 9] (see Materials and Methods) and with tuning curves shown in Figure 3A. We then define the “marginal SSI” of a neuron in the context of a population as the difference between the four-neuron population SSI and the SSI of the population of the remaining three neurons after the single neuron of interest is removed.

The SSI for the four-neuron population with the measured noise level is shown in Figure 3B (thin line). For this relatively low level of noise, there are eight peaks in the population SSI, located near the points of maximal slope of all four neurons. This population SSI is clearly related to the single neuron SSI of Figure 2B (thick line), with each neuron contributing two of the population peaks.

Figure 3C and 3D (thin lines) show the population SSI with 3× and 5× this noise level respectively. For these higher noise levels, the peak of the population SSI shifts to the points of intersection of the tuning curves, where the effect of noise is reduced by the cooperative encoding of the neighboring neurons. Even at 5× the noise level ( Figure 3D), the peaks in the population SSI do not transition to the locations of the individual tuning curve peaks.

In contrast, the location of the largest marginal SSI does exhibit a slope-to-peak transition, reflecting a change in the contribution of the individual neurons with increasing noise level. Consider the neuron centered at θ = 0 ( Figure 3A, bold). For low noise levels ( Figure 3B), the marginal SSI (thick line) is highest near the maximum slope of the tuning curve (line 1). The low marginal SSI near the tuning curve peak reflects the redundancy of the information encoded by the highlighted neuron with information encoded by neighboring neurons. This situation changes at higher noise levels: at 3× this noise level ( Figure 3C), the largest marginal SSI transitions to the location of the intersections of the tuning curves (line 2), and at 5× the noise level ( Figure 3D), the largest marginal SSI is at the peak of the tuning curve (line 3).

Thus, the marginal contribution of a single neuron to the information encoded by a population of neurons undergoes a transition from slope to peak but at a higher noise level than the same neuron considered in isolation (5× versus 3×), reflecting the effective reduction in the network noise level due to population averaging. In considering both the neuron in isolation ( Figure 2B) and its marginal contribution to the remainder of the population ( Figure 3), we describe two limits of its contribution to the population information about each stimulus. What is notable is that in both extremes, as noise increases, the maximum of the SSI transitions from the high-slope region of the tuning curve, where fine discrimination can be sensitively performed, to the peak of the tuning curve, where coarse discrimination is most important.

### Application to Discrimination Tasks

Thus far we have considered how well stimuli are encoded by a neuron in situations when all values of θ are equally probable. In such cases, we have shown that the degree of neuronal variability affects the relative importance of fine discrimination, which favors stimuli located in high-slope regions of the tuning curve, versus coarse discrimination which favors stimuli located near the tuning curve peak.

**Figure 4:**
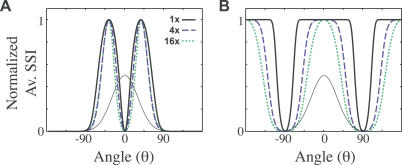
The Maximum SSI Is Independent of the Noise Level in Two-Alternative Discrimination Tasks (A) The average SSI of the neuron from Figure 1 for a discrimination task with two stimuli located at ±3° centered around the angle θ, for low-noise (solid) and high-noise (dashed) conditions. The dotted line shows the average SSI for 4× the high-noise condition, demonstrating that there is no transition from slope to peak. For reference, the tuning curve of the neuron is shown as a thin solid line. (B) The same neuron for a discrimination task with stimuli at +0° or +180° from θ in the three noise conditions mentioned.

The effects of these two types of discrimination can be isolated in the context of two-alternative discrimination tasks. In these tasks, the ability to discriminate between the two stimuli, *s*_1_ and *s*_2_, is related to the separation of their two corresponding response distributions *p*( *r*| *s*_1_) and *p*( *r*| *s*_2_). For fine discrimination tasks, these distributions will be separated in proportion to the slope of the tuning curve between the stimuli. As a result, pairs of stimuli located in regions of high slope will be more distinguishable than pairs located near the tuning curve peak. This is reflected in the average SSI ( Figure 4A) of the generic model neuron from Figure 1 during a fine discrimination task with stimuli located at ±3° from a central angle θ. The maximal SSI is at high-slope regions and does not change location with increasing noise level (low noise, solid line; high noise, dashed line). Although nearby stimuli become less distinguishable with increasing neuronal variability, they are always most distinct from each other in higher slope regions. To demonstrate the robustness of these results, the SSI for 4× the high-noise case is plotted as well (dotted line).

Another common discrimination experiment requires the subject to distinguish between opposite instead of nearby directions [ 4, 13]. Because most tuning curves are less than 180° in width, this implies that one of the two stimuli will always be located in the flat, non-tuned “background” portion of the tuning curve. Therefore, in contrast to the fine discrimination task above, the response distributions in this paradigm are most distinguishable when one of the stimuli is located near the peak of the tuning curve, and as a result its responses are separated from background firing as much as possible. This is reflected in the average SSI shown in Figure 4B, simulated for the same noise levels considered in Figure 4A. Again, neuronal variability does not cause a transition from peak to slope because fine discrimination of nearby stimuli is not relevant to the task, and neurons whose tuning curve peak is aligned with either of the two stimuli will always convey the most task-relevant information.

Note that where the SSI is flat, perfect discrimination occurs because, except in the case of very high noise levels, the two relevant response distributions are completely distinct. The degree of distinctiveness of high firing rates is more extreme in this context compared with the general case of “coarse discrimination” previously discussed, which factored in both discrimination from background firing rates as well as from responses evoked by stimuli on the flanks of the tuning curve. Experiments testing opposite-angle discrimination typically introduce additional noise [ 13] (see Protocol S1) to probe perceptual thresholds so that the relevant average SSI might look more like the highest noise example shown (dotted line).

In summary, applying the SSI to discrimination experiments demonstrates how experimental context can determine whether the slope or peak of the tuning curve is most relevant by presenting stimulus pairs that isolate each of the two competing effects: fine discrimination versus coarse discrimination. When both types of discrimination are relevant, as in the simulations of Figure 1, the neuron shown in Figure 4 exhibits a slope-to-peak transition under the same noise conditions. Thus, the absence of noise sensitivity in discrimination tasks is not necessarily indicative of more general experimental paradigms.

## Discussion

Previous studies have identified either stimuli at the maximum firing rate or maximum slope portion of a tuning curve as the best encoded by an individual neuron. Here, we demonstrate that either can be correct depending on the experimental context and level of neuronal variability. We show that the results of several information-based measures of stimulus encoding reduce to the intuition that stimuli can be well encoded for two separate, and often competing, reasons: either they are easily discriminated from nearby stimuli (at high-slope regions of the tuning curve) or they are associated with the most distinct responses (at the tuning curve peak). We refer to these two effects as “fine discrimination” and “coarse discrimination,” respectively. The amount of neuronal variability greatly influences the relative importance of these two types of discrimination, such that the best-encoded stimulus, in the sense of having the maximum SSI, can change systematically from a high-slope region of the tuning curve for low noise to the peak of the tuning curve for high noise.

We have chosen to use the SSI to illustrate this basic tradeoff between fine and coarse discrimination because it directly ties how well stimuli are encoded to the information of their underlying responses. As we demonstrate in Protocol S2, the results described above match those obtained with other information-based metrics of stimulus significance such as the transinformation [ 7, 11] and the local information [ 14]. Although the SSI was applied to relatively simple tuning-curve-based models here, its application can be extended to a wide-range of contexts including multi-dimensional tuning curves and timing-based studies of neural responses [ 6].

### Population Size Mitigates the Effect of Noise

The transition from maximum-slope to maximum-firing-rate encoding occurs both for an isolated neuron and for a neuron in the context of a population. However, the transition in the context of a population occurs at a higher noise level because the population effectively averages out uncorrelated noise. This is consistent with the results of studies of large populations that use the Fisher information and find maximal information at the high-slope regions of the tuning curve [ 2, 3]. While the SSI is computationally constrained to the analysis of small populations, the Fisher information does not provide accurate estimates of coding efficiency when the population size is below a noise-dependent threshold [ 15]. As a result, these measures allow complementary analyses of tuning curves that span the range of population sizes and likely agree in the limit of large population size [ 16].

Thus, a key factor in this debate is the relevant number of neurons participating in a perceptual decision. While this number may be strictly limited in invertebrate systems such as the cricket, entire regions of the medial temporal cortex may be relevant to perceptual decisions based on visual motion. However, in such cases, the effective population size may be much smaller due to the level of correlations between neurons, which could affect the ability to average out noise across neuronal populations [ 17– 20], and due to the relationship between the average neuronal firing rate and the time window over which stimuli are processed, which could affect the ability to temporally average out noise [ 21].

Experiments linking the performance of individual neurons to perceptual decisions bear directly on this issue [ 4, 5, 13, 22, 23]. They suggest that only a subset of neurons participate in perceptual decisions, namely those that have a direct relationship to the task due to their feature selectivity relative to the stimulus [ 4, 5], which may include selectivity to features that are irrelevant to the perceptual decision [ 23]. Some studies have estimated that as few as four neurons participate in a perceptual decision in the case of opposite angle discrimination [ 24], whereas other studies found that more than 20 [ 5] or 100 [ 25] neurons must be pooled to match behavioral performance. The discrepancy between these studies of the number of neurons participating in perceptual decisions could result from factors such as the difficulty of the task or the relevant integration window over which the neuronal and perceptual response is considered [ 5, 26].

### The Presence of a Slope-to-Peak Transition

We find that whether a given neuron contributes more information about the slope or peak of its tuning curve depends on the level of neuronal variability, the experimental context, and the amount of population pooling. Although the three information-based metrics that we have applied all robustly find this transition, it has not been observed in previous studies that use other metrics of stimulus encoding, such as ROC analysis [ 27], the neurometric function [ 5], the discrimination index [ 28], the Chernoff distance [ 8], and the Fisher information [ 3]. These other metrics all measure the ability of the neuron to discriminate between two stimuli, which, as we demonstrated in Figure 4, explicitly neglects the tradeoff between fine and coarse discrimination that is present in any context where more than two stimuli are relevant. (Note that, although the Fisher information is not explicitly a discrimination measure, it only applies to the large-population limit where, as demonstrated with the SSI in Figure 3 and discussed at greater length in Protocol S2, fine discrimination is favored.) In this light, it is possible to interpret our results as reflecting the ability to discriminate a given stimulus from all others in the ensemble, which is explicitly measured by the local information [ 14] (see Protocol S2) that gives very similar results to the SSI.

The focus of previous metrics on two-alternative discrimination likely reflects the fact that most experiments attempting to link single neuron encoding to perception and behavior have used discrimination tasks to do so [ 29]. In this sense, our results suggest that in an experimental task with more than two relevant directions, the neurons contributing most to perception may depend on the amount of neuronal variability and the size of the relevant pool of neurons participating. Conversely, experimental manipulation of neuronal variability (as, for example, described in Protocol S1) may be able to directly test for a slope-to-peak transition, and the location of such a transition might reflect both the size of the population involved and the relevant time window of underlying coding.

Given our results, it is interesting to speculate whether a system's encoding strategies change depending on the stimulus strength, state of attention, or other task specific factors such as the reward level [ 30] that could affect the level of neuronal variability. This could involve a dynamic switch in the neural populations monitored [ 5, 23], as discussed above, or a change in the shape of the tuning curves themselves with changes in stimulus properties [ 31].

Although tuning curves describe which stimuli cause a neuron to fire the most, they do not reveal which stimuli are best encoded. The analysis method presented here, by combining average firing rate and the associated response variability into a single metric, allows tuning curves to be interpreted in terms of which stimuli lead to the most informative neuronal responses. The method can be applied to a wide variety of stimulus contexts and provides a unified framework for interpreting a diverse set of data ranging from two-alternative discrimination tasks to multi-alternative encoding paradigms. As we have shown, such analyses can reveal effects of neuronal variability on sensory encoding that are not obvious from the examination of tuning curves alone.

## Materials and Methods

The SSI and other measures are calculated for several examples: a “generic” tuning curve with features common to many sensory neurons, a visual neuron orientation tuning curve adapted from the recordings of Kang et al. [ 8], and a cricket cercal neurons fit from the measurements of Miller et al. [ 9].

For all tuning curve examples, the firing rate of each neuron was fit as *r*(θ) = [ *f*(θ) + *η*] _+_ where [ ] _+_ denotes rectification of negative values to zero, *f*(θ) denotes the “tuning curve” function before the addition of noise, and *η* denotes Gaussian noise with zero mean and standard deviation *σ*. For the cricket cercal tuning curve example, parameters were taken from the measurements of Miller et al. [ 9] with *f*(θ) = [cos(θ − θ _0_) − 0.14]/0.86, and *σ* = *A*[0.048 + 0.052 *f*(θ)]. Here, θ _0_ is the wind direction value that elicits the maximum average firing rate and differs by 90° for each of the four neurons. We refer to the values reported in Miller et al. [ 9] as the low-noise case, corresponding to *A* = 1. For the high-noise cases, *A* = 3 or 5. Note that correlations between neurons were not reported in these experiments; therefore we model neuronal responses as conditionally uncorrelated when considering the population responses. However, since our methods can be applied to arbitrarily specified joint probability distributions, they can more generally account for any measured correlations between responses.

For the tuning curve example of Figures 1 and 4, *f*(θ) was taken to be a Gaussian with a standard deviation of 30° centered at the orientation θ = θ _0_, with the same noise model described above, although with *A* = 0.5 for the low-noise case and *A* = 2 for the high-noise case. This choice of noise model was made for simplicity and for consistency with the simulations of Figure 2B and did not affect our qualitative results. As we demonstrate in Protocol S1, a transition from slope to peak robustly occurs for a wide range of noise models; examples include when the background noise or stimulus-dependent noise are separately manipulated, when noise is determined by Poisson statistics, and when an additional source of noise arising from uncertainty in the stimulus is considered.

For the orientation-tuned neuron of Figure 2A, we used mean values for V1 neurons reported in Kang et al. [ 8], corresponding to a Gaussian tuning curve with a standard deviation of 22.2° and a baseline response equal to 16% of the peak. The mean number of spikes produced at the tuning curve peak was 39, and the noise as a function of θ obeyed a Poisson distribution about the mean spike count. In the examples shown, we display results for neurons with no explicit direction selectivity. This choice was made for simplicity and did not qualitatively change our results.

The SSI was computed numerically from the joint probability distribution *p*( *r*,θ) = *p*( *r*|θ) *p*(θ) where *p*( *r*|θ) was obtained from the noise model. For Figures 1– 3, *p*(θ) was a uniform distribution. For Figure 4, *p*(θ) corresponded to a discrete distribution with value 0.5 for each of the two alternatives. For all simulations, *r* and θ were discretized into bins of size 0.01 Hz and 1°, respectively, for the single-neuron results and 0.06 Hz and 5°, respectively, for the population results. At this fine resolution, the results did not depend on the bin size.

Note that in the single neuron case, a particular firing rate might be associated with stimuli either on the left or right side of the tuning curve with equal probability. This ambiguity does not affect any of the information measures used in this paper, since it contributes one bit to both the stimulus entropy and the response-conditional entropy, which cancels in the information measures. As a result, measurements of the SSI (as well as those used in the Supporting Information) are identical to the situation where only half a tuning curve is considered.

For the two-alternative discrimination task ( Figure 4), the average SSI is plotted rather than the two individual SSI's (which closely match) because comparisons between angles in this figure do not measure how well a given angle is encoded within a larger stimulus ensemble (as in previous figures), but rather how well the ensemble itself is encoded by neural responses. In this case, the average SSI is exactly equal to another information-based measure of stimulus encoding, the local information [ 14] (see Protocol S2), as well as the mutual information between the neuron's responses and the two stimuli [ 6].

## Supporting Information

Protocol S1 The Slope-to-Peak Transition Is Robust to the Noise Model (234 KB PDF)

Protocol S2 Information Measures of Stimulus Encoding (428 KB PDF)

## Abbreviations

MT: medial temporal
SSI: stimulus-specific information
