# Supplementary material for: Tuning Curves, Neuronal Variability, and Sensory Coding
Source: PLoS Biol. 2006 Mar 21;4(4):e92. doi: 10.1371/journal.pbio.0040092 (PMC1403159; doi:10.1371/journal.pbio.0040092)
Supplement: Supplementary file 1 [file pbio.0040092.sd001.pdf]

Supporting Information S1: The Slope-to-Peak Transition Is Robust to the Noise Model

(*accompanying Butts and Goldman, PLoS Biology, 2006*)

The purpose of this supplemental material is both to illustrate the robustness of the transition to several different representative noise models for single neurons and to provide concrete examples demonstrating the application of the stimulus-specific information (SSI) to tuning curves.

Throughout our paper, we define the "neuronal variability" of the response r by the probability distribution $p(r|\theta)$, which is the distribution of responses of a neuron given repeated presentations of the same stimulus θ. Due to this general formulation, many factors can contribute to neuronal variability such as intrinsic neural noise, integration time of the neural response, and aspects of the stimulus not explicitly represented by the tuning curve. Notably, this simple formulation of neuronal variability is relatively easy to characterize experimentally, and for example can be coarsely estimated from the same data used to measure the tuning curve itself.

Although we cannot test every possible noise model, we have found in all cases tested that a slope-to-peak transition occurred. While the source of variability determines the fine structure of the distributions $p(r|\theta)$ and thus affects the precise noise level for which a transition occurs, it was not found to affect the existence of a transition. Below, we show simulations for several common examples of noise models and identify the parameter values at which the slope-to-peak transition occurred. The particular noise models considered are: a linear dependence of the noise on the neuronal firing rate, a Poisson noise model (with approximately square root dependence of the noise on firing rate), and a case in which the noise is directly driven by variability in the stimulus (as in the random dot motion experiments of Britten et al., 1992).

Linear Model of Neuronal Noise

The most straightforward characterization of the stimulus-conditional response distribution $p(r|\theta)$ is by its mean and standard deviation. The mean of this distribution is the value of the tuning curve $f(\theta)$ itself, and $p(r|\theta)$ is typically modeled with a Gaussian distribution. [Note that distributions were truncated at zero firing rate; this was verified to have negligible impact on results.]

For example, Miller et al. (1991) report the standard deviation of the noise in their system as a stimulus-independent term combined with a stimulus-dependent term proportional to the tuning curve itself:

$$\sigma(\theta) = \alpha + \beta f(\theta)$$

In the main text of the paper, we consider the effect of varying α and β simultaneously. Here we consider the effect of varying α and β separately.

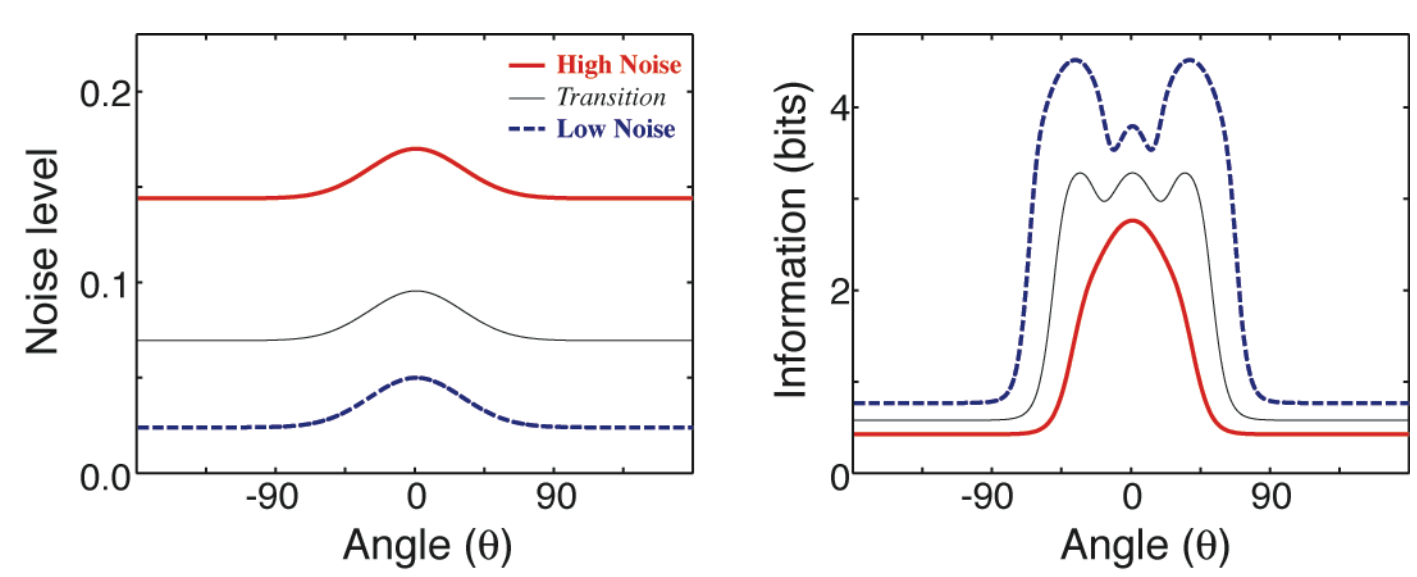

Figure S1-1: The noise as a function of stimulus θ when the noise background is varied independently. **Left**: The noise as a function of θ for low noise (*blue dashed line*), the transition noise level (*thin solid line*), and the high noise case (*red solid line*). **Right**: The SSI for these three cases.

First, we show the effect of varying the background level α while holding β constant at the value β=0.026. Figure S1-1 shows a "low noise" case (*red solid line*) with α_L = 0.024 (equivalent to the low noise case in Figure 1

of the main text) and a "high noise" case (*blue dashed line*) with α_H = 0.144 (6 times α_L). The background noise level where the high-slope region and the peak of the tuning curve have the same SSI occurs for α_{TR}=0.070 (*thin black line*). Note that changing the background firing rate alone has the effect of shrinking the region of the tuning curve that conveys significant SSI (*i.e.*, the width of the high-noise SSI is smaller compared to the width of the low-noise SSI).

Next, in Figure S1-2 below, we show the effect of varying the stimulus-dependent component β of the noise while the background was held constant at the value α= 0.024. The low noise case again corresponds to Figure 1 in the main text (β_L = 0.026). The high noise case is shown for β_H=0.42, and the transition occurs at β_{TR}=0.11.

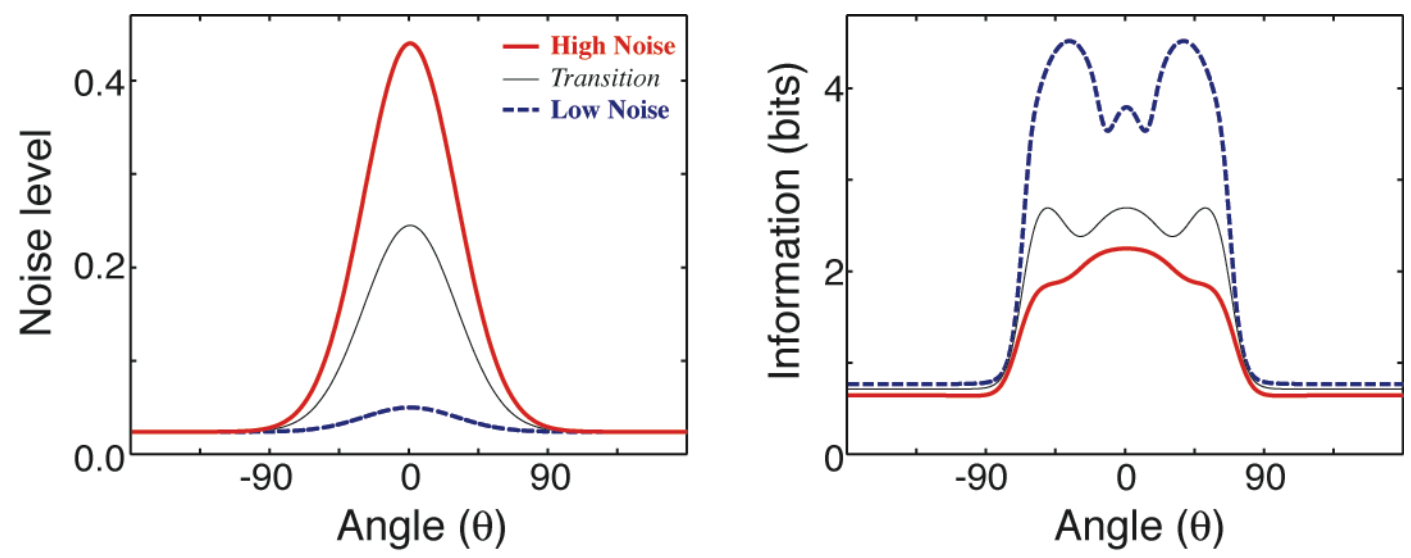

Figure S1-2: The noise as a function of stimulus θ when the stimulus-dependent noise is varied independently. **Left**: The noise as a function of θ for low noise (*blue dashed line*), the transition noise level (*thin solid line*), and the high noise case (*red solid line*). **Right**: The SSI for these three cases.

In this case, the width of the SSI curve does not decrease with increasing noise (as it did in Fig S1-1). Furthermore, the noise level at which a transition occurs is higher in this example than when the background noise is manipulated, because increasing the stimulus-dependent noise disproportionately increases the variability at the peak, resulting in smaller SSI values at the peak.

In most situations, the background rate and stimulus-dependent rate vary simultaneously. For example, in Figures 1 and 4 of the main text, α and β are varied proportionally. In this case, a transition occurs at α_{TR}=0.058 and β_{TR}=0.063 (Figure S1-3; the corresponding low and high noise values shown in the main text figures are α_L=0.024; β_L=0.026; α_H=0.096; β_H=0.104).

Altogether, these simulations illustrate that the slope-to-peak transition is a robust phenomenon that can occur over a wide range of parameters.

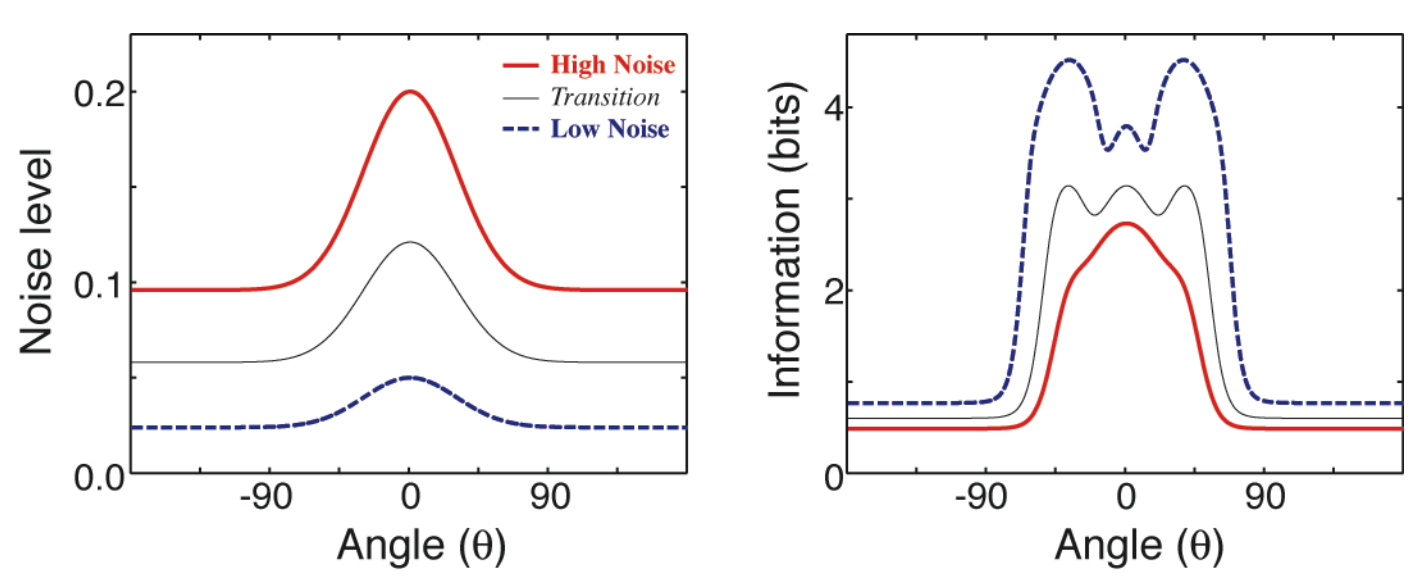

Figure S1-3: The noise as a function of stimulus θ when both terms are proportionally varied. **Left**: The noise as a function of θ for low noise (*blue dashed line*), the transition noise level (*thin solid line*) and high noise case (*red solid line*). **Right**: The SSI for these three cases. The high and low noise levels are the same as those shown in Figures 1 and 4 (main text).

Poisson Noise

In the above examples, the stimulus-dependent component of the noise was chosen to be linear both for simplicity and to match the experimental findings of Miller et al. (1991). However, since increasing the variability at the peak pushes the slope-to-peak transition to higher noise levels (Figure S1-2), proportional scaling of the variability with mean rate is one of the least effective ways to drive a transition.

For noise based on Poisson distributions, the stimulus-dependent term is approximately proportional to $\sqrt{f(\theta)}$. As a result, there is proportionally less noise at the peak and it is easier to see a transition. We found that a neuron with mean properties of the V1 data of Kang et al. (2004) is already in the "high noise" regime (Figure 2A, main text). Increasing the firing rate or the integration time window reduces the noise enough that the best-encoded stimulus transitions to regions of high slope. Figure S1-4 illustrates the transition that occurs in this neuron when the number of spike counts is lowered. The low-noise case corresponds to an average spike count of 154 spikes at the tuning curve peak, the high noise case corresponds to 39 spikes, and the transition occurs at 95 spikes.

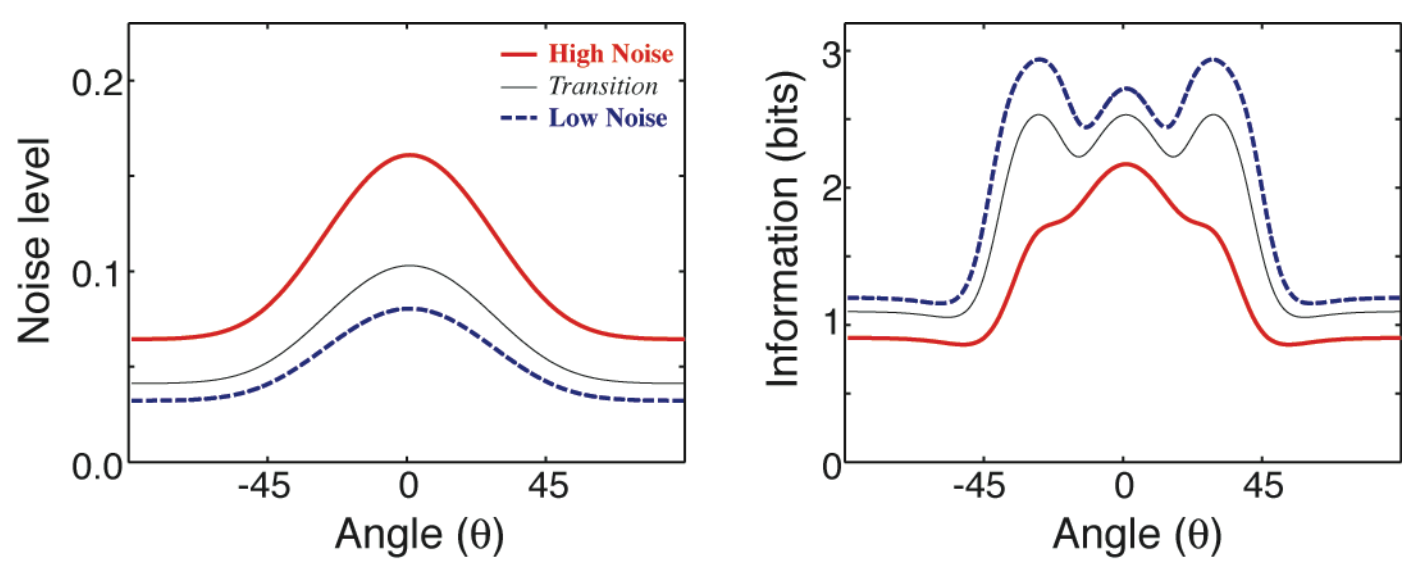

Figure S1-4: The noise as a function of the stimulus θ for Poisson variability. **Left**: The noise as a function of θ for low noise (*blue dashed line*), the transition noise level (*thin solid line*), and the high-noise case (*red solid line*). **Right**: The SSI for these three cases. The high-noise case shown here is the same as the "average rate" V1 neuron considered in Figure 2A (main text).

Noise in the Stimulus

Introducing "noise" into the stimulus itself can increase the variability of the neuronal response to a given stimulus parameterized by θ. For example, as mentioned in the main text, Britten et al. (1992) use multiple targets moving in randomly oriented directions to make an opposite-angle discrimination task perceptually difficult. In such cases, neuronal variability might be simply modeled as the variability of the neuron's response to single targets $p_0(r|\theta)$ (like that discussed above) combined with the distribution of target directions parameterized by θ, which includes several different targets occurring with probabilities $p(\theta'|\theta)$. In this case, the total neuronal variability is given by:

$$p(r|\theta) = \sum_{\theta'} p_0(r|\theta')\, p(\theta'|\theta)$$

In the experiments of Britten et al., $p(\theta'|\theta)$ takes the form of a delta-function at the preferred stimulus direction plus uncorrelated (background) stimuli chosen from a uniform distribution, *i.e.*, $p(\theta'|\theta) = A\,\delta(\theta'-\theta)+B$. In this case, the resulting neuronal variability is given by

$$p(r|\theta) = A\, p_0(r|\theta) + B$$

so that the background increases in proportion to the fraction of uncorrelated target stimuli (parameterized by B). Thus, increasing the number of uncorrelated targets simply increases the background noise, driving a transition like that shown in Figure S1-1. More general cases of stimulus noise can be numerically solved using the above framework.

References

Britten KH, Shadlen MN, Newsome WT, Movshon JA (1992) The analysis of visual motion: a comparison of neuronal and psychophysical performance. J Neurosci 12:4745-65.

Kang K, Shapley RM, Sompolinsky H (2004) Information tuning of populations of neurons in primary visual cortex. J Neurosci 24: 3726-3735.

Miller JP, Jacobs GA, Theunissen FE (1991) Representation of sensory information in the cricket cercal sensory system. I. Response properties of the primary interneurons. J Neurophysiol 66: 1680-1689.
