# Supplementary material for: Tuning Curves, Neuronal Variability, and Sensory Coding
Source: PLoS Biol. 2006 Mar 21;4(4):e92. doi: 10.1371/journal.pbio.0040092 (PMC1403159; doi:10.1371/journal.pbio.0040092)
Supplement: Supplementary file 2 [file pbio.0040092.sd002.pdf]

Supplemental Information S2: Information Measures of Stimulus Encoding

(*accompanying Butts and Goldman, PLoS Biology, 2006*)

Several information-based measures of how well stimuli are encoded by neural responses exist in the literature. In the main text, we describe results obtained using the *stimulus-specific information* (SSI) (Butts, 2003). Below, we compare this result to those obtained with three additional information-based measures: (1) the *transinformation* proposed by Theunissen and Miller (1991) and later renamed as *the specific surprise* by DeWeese and Meister (1999); (2) the *local information* proposed by Bezzi et al. (2002); and (3) the *Fisher information*, used by a variety of studies including several that specifically address the issue of tuning curves and information (Seung and Sompolinsky, 1993; Salinas and Abbott, 1994; Brunel and Nadal, 1998; Pouget et al., 1999; Zhang and Sejnowski, 1999; Bethge et al., 2002; Xie, 2002).

Below we apply these measures to the generic tuning curve example of Figure 1. These results are representative of all tuning curves examples considered in the main text (including those based on experimental characterizations considered in Figure 2).

Transinformation (Theunissen and Miller, 1991) and **Specific Surprise** (DeWeese and Meister, 1999)

Transinformation was proposed originally as a measure of how well different wind directions are encoded by interneurons in the cricket cercal system (Theunissen and Miller, 1991). It was later re-introduced under the name *specific surprise* and used as an alternative to *specific information* in associating a mutual-information-based measure with particular stimuli and responses (DeWeese and Meister, 1999). It is defined as

$$i_{sur}(\theta) = \sum_r p(r \mid \theta) \log_2 \frac{p(r \mid \theta)}{p(r)}$$

Like the specific information and the SSI discussed in this paper, the average specific surprise across the stimulus ensemble is the mutual information: thus it can be viewed as a measure of the contribution of each stimulus to the total information. The particular interpretation of the specific surprise as a measure of the information associated with particular stimuli is explicitly compared to that of the specific information and SSI in Butts (2003).

The specific surprise for the tuning curve example of Figure 1 (main text) is shown in Figure S2-1 below for the low and high noise cases:

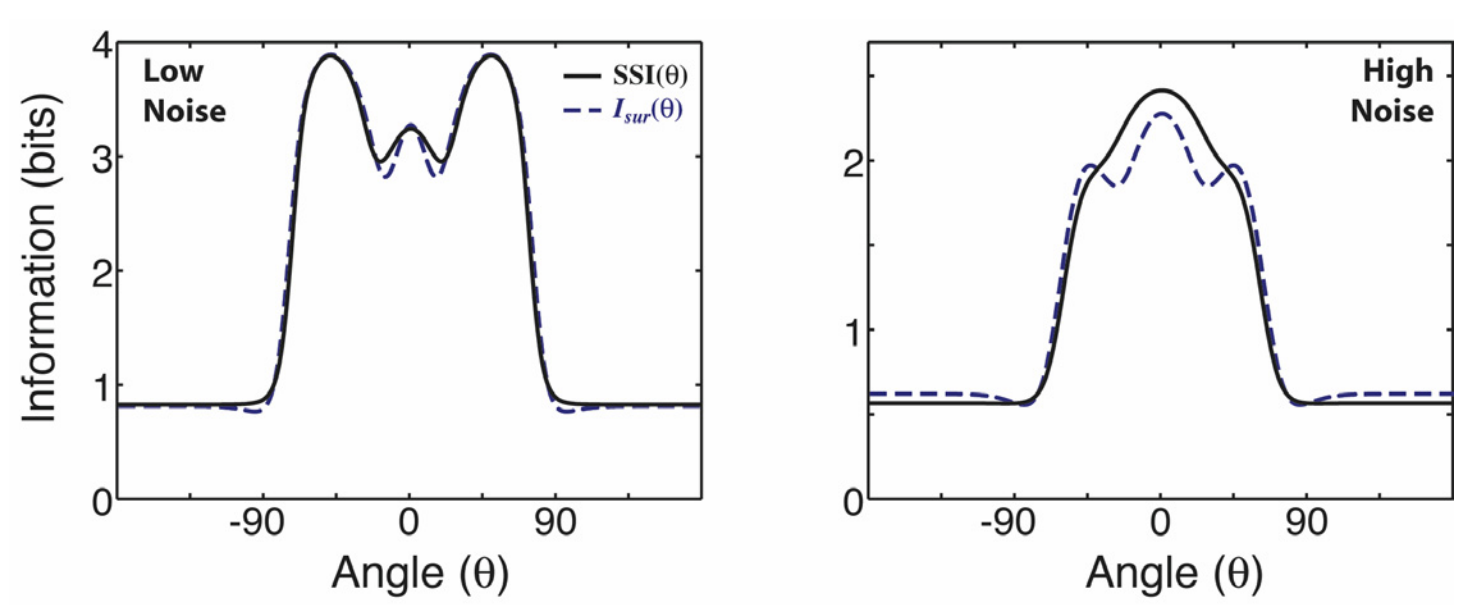

Figure S2-1: Comparison of the specific surprise and SSI for the tuning curve example described in Figure 1 for low and high noise cases.

While there are subtle differences between the information assigned to stimuli by the SSI (*solid*) and specific surprise (*dashed*), these two measures are in close agreement.

The transinformation in the low noise case (*left*) was originally published in the study of Theunissen and Miller (1991), although the effects of changing the noise level in the system were not investigated.

Local information (Bezzi et al., 2002)

The *local information* was proposed by Bezzi et al. (2002) to provide a measure of the amount of information encoded by place cells in various regions of the hippocampus about an animal's location. It is defined as the mutual information between the response of a neuron and whether or not a particular stimulus θ is present. Specifically, it is defined as:

$$i_{loc}(\theta) = p(\theta)\sum_r p(r \mid \theta)\log_2 \frac{p(r \mid \theta)}{p(r)} + p(\sim \theta)\sum_r p(r \mid\sim \theta)\log_2 \frac{p(r \mid\sim \theta)}{p(r)}$$

where $p(\sim \theta) = \sum_r p(\sim \theta \mid r)p(r)$ and $p(r \mid\sim \theta)p(\sim \theta) = \sum_{\theta' \neq \theta} p(r \mid \theta')p(\theta')$. Note that if $p(\theta)$ is small, the expression in the logarithm of the second term is close to one, and the second term barely contributes to $i_{loc}(\theta)$. Thus, as described in Bezzi et al. (2002), in the limit of small $p(\theta)$, $i_{loc}(\theta) \approx p(\theta)\, i_{sur}(\theta)$. This limit applies in the tuning curve examples considered in this paper so that the local information is nearly identical to the specific surprise (compare Figures S2-1 and S2-2). As a result, the local information also closely matches the SSI.

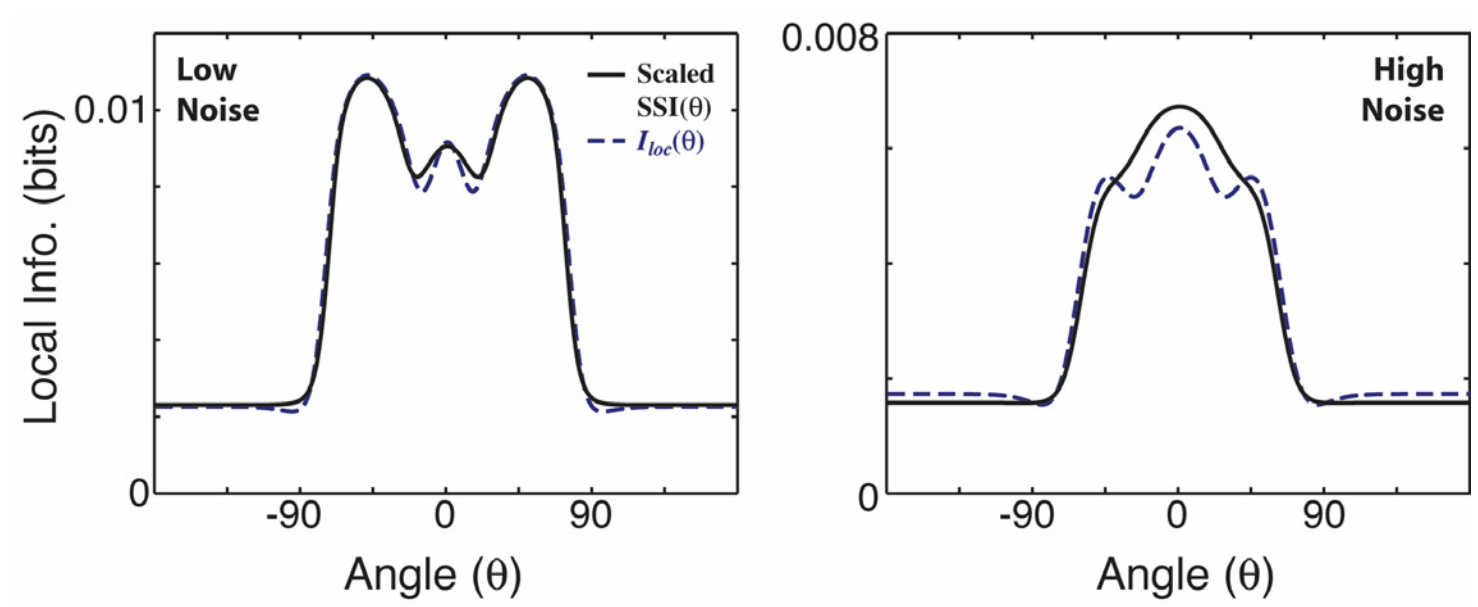

Figure S2-2: Comparison of the local information and SSI for the tuning curve example described in Figure 1 (main text) for low and high noise cases. Here the SSI is scaled by $p(\theta) = 1/360$ for a direct comparison with the local information.

Fisher information (*e.g.*, Seung and Sompolinsky, 1993)

The *Fisher information* has been used in numerous studies of tuning curves (Seung and Sompolinsky, 1993; Salinas and Abbott, 1994; Brunel and Nadal, 1998; Pouget et al., 1999; Zhang and Sejnowski, 1999; Bethge et al., 2002; Xie, 2002), because it is both an explicit stimulus-specific measure and easily applied to large populations of neurons. The Fisher information $J(\theta)$ is defined as:

$$J(\theta) = \left\langle \left[\frac{\partial}{\partial \theta} \log\, p(r \mid \theta) \right]^2 \right\rangle_{p(r|\theta)}$$

where $p(r|\theta)$ is the distribution of responses for a given stimulus parameter (*i.e.*, the neural variability as discussed throughout this study) and $\langle \cdot \rangle$ represents an average over these responses. The meaning of the Fisher information relates to the specific problem of decoding the stimulus from a neural response: $1/J(\theta)$ sets a lower bound on the variance of any unbiased estimate of θ given the response distribution $p(r|\theta)$ (through the Cramer-Rao inequality). In this way, Fisher information is a measure of how well a particular angle can be decoded given the variability of the neural response.

We solve analytically for $J(\theta)$ for the bell-shaped tuning curve (Figure 1A of main text) with mean firing rate $f(\theta)$ and response variability given by $\sigma(\theta) = a + bf(\theta)$ (with the values of a and b given in the Methods). It is necessary in this case to allow for negative firing rates, which were verified to have an insignificant effect on the results of the other measures discussed (and thus are directly comparable to the measures described above). In this case, the Fisher information is given by:

$$J(\theta) = \left(\frac{1+2b^2}{(\Delta\theta)^4} \right) \frac{\theta^2 [f(\theta)]^2}{[a + bf(\theta)]^2}$$

It is shown in comparison to the SSI in Figure S2-3 (where the SSI is scaled so that its mean value across angles matches that of the Fisher information).

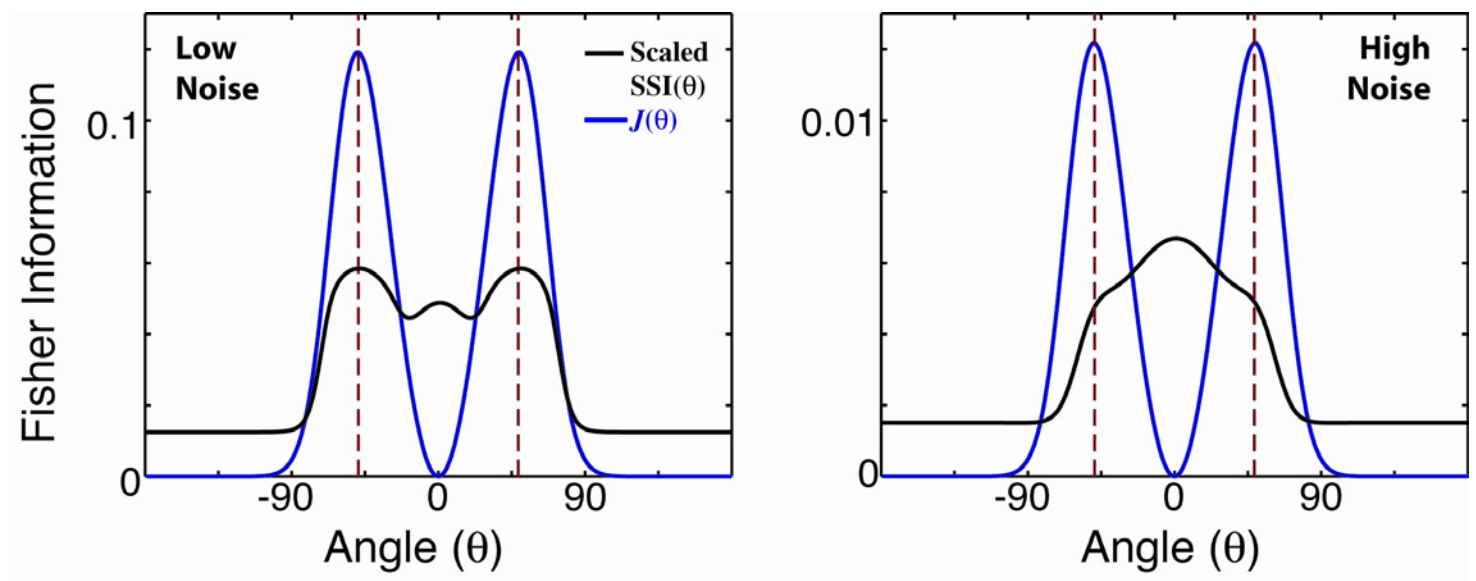

Figure S2-3: Comparison of the Fisher information and SSI for the tuning curve example of Figure 1 (main text) for low and high noise cases. Here the SSI is scaled such that the mean SSI is equal to the mean $J(\theta)$.

The peaks of the Fisher information and the SSI are in close agreement for the low noise case (*left*), although notably the Fisher information is zero at the peak of the tuning curve whereas the SSI at the peak is almost as large as the SSI at the maxima. In contrast, despite the fact that neural variability is explicitly incorporated into the definition of Fisher information, the locations of the maximum values of Fisher information in this example are insensitive to noise level, although the overall magnitude of the Fisher information is scaled by the squared magnitude of the noise.

This discrepancy between $J(\theta)$ and all other measures discussed here reflects the locality of the Fisher information metric, since it only takes into account the discriminability of neighboring stimuli and neglects the fact that many stimuli might evoke the same response (Kang et al., 2004; Dayan and Abbott, 2001). This is related to the surprising result that the Fisher information is zero at the tuning curve peak (which implies that the lower bound of an unbiased estimator is ∞): although high firing rates are useful at distinguishing the subset of stimuli around the peak of the tuning curve from other stimuli ("coarse discrimination"), high firing rates are nearly useless at discriminating between neighboring stimuli at the peak ("fine discrimination").

However, as more neurons are used to produce an estimate of θ, the subset of stimuli that evoke the same multi-dimensional response becomes increasingly localized. Thus, in the limit of large populations with uniformly distributed tuning curves, the contribution of a single neuron's response becomes completely local. This is reflected in the SSI of the 4-neuron population where, in the low noise case (Fig. 3B), the marginal SSI of a single neuron all but disappears at the peak of its tuning curve, and the transition of stimuli with the highest SSI from high-slope to peak occurs at a higher noise level.

As discussed in the main text, the Fisher information and the SSI are useful in different limits of population size. The application of the SSI and the other Shannon-based measures discussed above is computationally limited to small populations, although analytic approximations may be applicable (Brunel and Nadal, 1998). At the same time, recent studies (Bethge et al., 2002; Xie, 2002) have detailed the lack of applicability of Fisher information when the population size is smaller than a noise-dependent threshold (Xie, 2002). With regard to this issue, it is important to note that the presence of noise correlations may raise this threshold significantly (Zohary et al., 1994; Sompolinsky et al., 2001; Averbeck and Lee, 2004).

References

Averbeck BB, Lee D (2004) Coding and transmission of information by neural ensembles. Trends Neurosci 27:225-230.

Bethge M, Rotermund D, Pawelzik K (2002) Optimal short-term population coding: when Fisher information fails. Neural Comput 14:2317-2351.

Bezzi M, Samengo I, Leutgeb S, Mizumori SJ (2002) Measuring information spatial densities. Neural Comput 14:405-420.

Brunel N, Nadal JP (1998) Mutual information, Fisher information, and population coding. Neural Comput 10:1731-1757.

Butts DA (2003) How much information is associated with a particular stimulus? Network 14:177-187.

Dayan P, Abbott LF (2001) Theoretical Neuroscience. Cambridge, MA: MIT Press.

DeWeese MR, Meister M (1999) How to measure the information gained from one symbol. Network 10:325-340.

Kang K, Shapley RM, Sompolinsky H (2004) Information tuning of populations of neurons in primary visual cortex. J Neurosci 24:3726-3735.

Pouget A, Deneve S, Ducom JC, Latham PE (1999) Narrow versus wide tuning curves: What's best for a population code? Neural Comput 11:85-90.

Salinas E, Abbott LF (1994) Vector reconstruction from firing rates. J Comput Neurosci 1:89-107.

Seung HS, Sompolinsky H (1993) Simple models for reading neuronal population codes. Proc Natl Acad Sci U S A 90:10749-10753.

Sompolinsky H, Yoon H, Kang K, Shamir M (2001) Population coding in neuronal systems with correlated noise. Phys Rev E 64:051904.

Theunissen FE, Miller JP (1991) Representation of sensory information in the cricket cercal sensory system. II. Information theoretic calculation of system accuracy and optimal tuning-curve widths of four primary interneurons. J Neurophysiol 66:1690-1703.

Xie X (2002) Threshold behaviour of the maximum likelihood method in population decoding. Network 13:447-456.

Zhang K, Sejnowski TJ (1999) Neuronal tuning: To sharpen or broaden? Neural Comput 11:75-84.

Zohary E, Shadlen MN, Newsome WT (1994) Correlated neuronal discharge rate and its implications for psychophysical performance. Nature 370:140-143.
